# TCR activation kinetics and feedback regulation in primary human T cells

**DOI:** 10.1186/1478-811X-11-4

**Published:** 2013-01-14

**Authors:** Mateusz Poltorak, Boerge Arndt, Bhavani S Kowtharapu, Amarendra V Reddycherla, Vanessa Witte, Jonathan A Lindquist, Burkhart Schraven, Luca Simeoni

**Affiliations:** 1 Institute of Molecular and Clinical Immunology, Otto-von-Guericke University, Leipziger Str. 44, 39120, Magdeburg, Germany

**Keywords:** TCR-mediated signaling, Feedback regulation, Cell-fate specification, T-cell activation, Lck, Erk, Signaling dynamics, CD4 crosslinking

## Abstract

**Background:**

Signaling through the TCR is crucial for the generation of different cellular responses including proliferation, differentiation, and apoptosis. A growing body of evidence indicates that differences in the magnitude and the duration of the signal are critical determinants in eliciting cellular responses.

**Results:**

Here, we have analyzed signaling dynamics correlating with either unresponsiveness or proliferation induced upon TCR/CD28 ligation in primary human T cells. We used two widely employed methods to stimulate T cells *in vitro*, antibodies either cross-linked in solution (sAbs) or immobilized on microbeads (iAbs). A comparative analysis of the signaling properties of iAbs and sAbs revealed that, under proliferation-inducing conditions, feedback regulation is markedly different from that leading to an unresponsive state. In fact, upon iAbs stimulation TCR-mediated signaling is prolonged by a positive feedback loop involving Erk, whereas sAbs strongly activate inhibitory molecules that likely terminate signaling. We additionally found that, by enhancing the phosphorylation of Src family kinases under proliferation-inducing conditions, signaling and T-cell activation are terminated.

**Conclusions:**

In summary, our analysis documents TCR signaling kinetics and feedback regulation under conditions of stimulation inducing either unresponsiveness or proliferation.

## Background

Ligation of the T cell receptor (TCR) triggers intracellular signals which may result in the initiation of markedly different cellular programs leading to differentiation, activation, survival, or apoptosis of T cells. One of the major questions in cell biology is how the activation of the same canonical signaling cascades dictates distinct biological outcomes. How signals are interpreted and translated into specific cellular outcomes has been extensively studied in PC-12 cells [1,2]. In these cells, it appears that differences in the magnitude and the duration of the Erk signal are critical determinants in eliciting the cellular response. For example, sustained Erk activation upon NGF treatment causes differentiation of the PC-12 cell line, whereas transient Erk activation upon EGF stimulation induces proliferation in the same cells [3]. Further studies have shown that NGF and EGF elicit different feedback regulation of the Ras-Erk cascade, which in turn results in distinct temporal profiles of Erk activity [4]. Whereas EGF triggers a negative feedback shutting off Raf-1 activity, NGF stimulation induces a positive feedback regulation of Raf-1. Differences in the duration of Erk activity are sensed by downstream transcription factors, thus altering the expression of specific genes required to carry out the cellular responses [5].

A similar dynamic behavior of Erk activity seems to exist also in thymocytes where strong and transient Erk activation induces apoptosis, whereas moderate but sustained Erk activity induces differentiation of immature T cells [6] and CD8^+^ TCR transgenic T cells [7]. How the dynamics of Erk activation is regulated in mature T cells is not yet clear.

Here, we have used primary human T cells to analyze TCR activation kinetics and feedback regulation. T cells were stimulated with CD3 and CD28 antibodies either cross-linked in solution (sAbs) or immobilized on microbeads (iAbs), which are two commonly used methods to study T-cell activation. Stimulation with sAbs induces only a transient signal and an abortive T-cell response resulting in unresponsiveness, whereas stimulation with iAbs induces sustained activation of the Erk cascade and cell proliferation [8]. We provide evidence that feedback regulation under proliferation-inducing conditions is markedly different from that leading to unresponsiveness. We demonstrate that sAbs activate negative feedback loops that terminate TCR-mediated signaling, whereas stimulation with iAbs results in the establishment of a positive feedback loop involving Erk-mediated phosphorylation of Lck prolonging TCR-mediated signaling. Collectively, our analysis provides novel insights into the regulation of the dynamic behavior of the TCR signaling module that controls cell-fate specification in primary human T cells.

## Results

### Sustained TCR-mediated signaling correlates with proliferation, whereas transient signaling parallels with unresponsiveness

**Figure 1:**
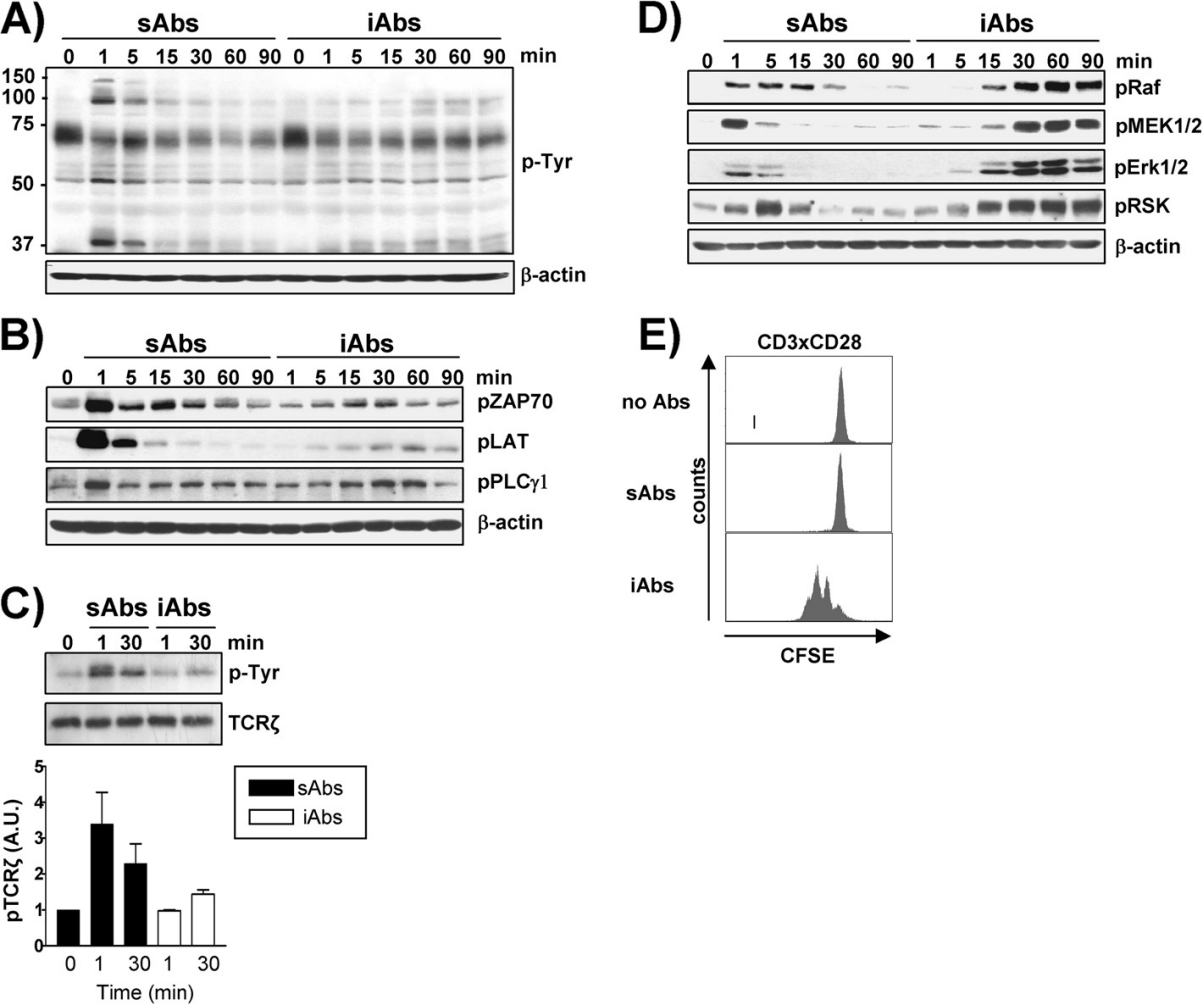
**Analysis of the signaling signature and functional effects of sAbs and iAbs stimulation. A-D)** Purified human T cells were treated with either soluble (sAbs) or immobilized (iAbs) CD3xCD28 mAbs for the indicated time periods. Total cell lysates **(A, B,** and **D)** or TCRζ immunoprecipitates **C)** were prepared and analyzed by Western blotting using the indicated Abs. One representative immunoblot of at least 3 independent experiments is shown. The phosphorylation of TCRζ was quantified using the 1D ImageQuant software and the values were normalized to the corresponding total TCRζ signal. Data represent the mean of the phosphorylation levels shown as arbitrary units ± SEM of 3 independent experiments. **E)** T cells were labeled with CFSE and stimulated as indicated. Proliferation was assessed after 72h by analyzing CFSE content on a FACS Calibur. One representative experiment of three independent experiments is shown.

Peripheral human T cells were stimulated using sAbs or iAbs. These stimuli induce markedly different activation kinetics and cellular responses (Figure 1). Stimulation with sAbs resulted in a strong and transient induction of global tyrosine phosphorylation, as well as of ZAP70, LAT, and PLCγ-1. In contrast, when primary human T cells were treated with iAbs, global tyrosine phosphorylation and the phosphorylation of ZAP70, LAT and PLCγ-1 were weak, but sustained (Figure 1A, B). Additionally, phosphorylation of TCRζ was greatly and rapidly enhanced upon sAbs. In contrast, iAbs stimulation induces only weak TCRζ phosphorylation (Figure 1C). Interestingly, the phosphorylation kinetics of PAG/Cbp, a transmembrane adaptor protein running at about 70 KDa which is dephosphorylated upon TCR stimulation [9], is comparable under both stimulation conditions (Figure 1A). We next analyzed the signaling kinetics of the Erk cascade. Surprisingly, we found that Erk was very strongly activated under both conditions of stimulation. However, the activation induced by iAbs was sustained and lasted up to 90 minutes whereas, upon stimulation with sAbs Erk activation was transient, peaked at 1–5 minutes, and rapidly declined thereafter (Figure 1D). Thus, despite the weak activation of proximal signaling molecules, iAbs are capable of inducing strong and prolonged activation of downstream signaling pathways.

It is generally accepted that transient signals triggered by soluble antibodies cannot induce productive T-cell responses. Indeed, we and others have demonstrated that human peripheral T cells, mouse OT-I transgenic T cells, and cytotoxic T-lymphocyte clones are not activated and do not differentiate upon stimulation with antibodies cross-linked in suspension [7,8,10]. Here, we have stimulated primary human T cells with either sAbs or iAbs and analyzed their functional responses. Treatment with sAbs failed to induce T-cell proliferation (Figure 1E). Conversely, Figure 1E shows that treatment of T cells with iAbs led to a strong proliferative response.

### Transient signaling is regulated via negative regulatory feedbacks

**Figure 2:**
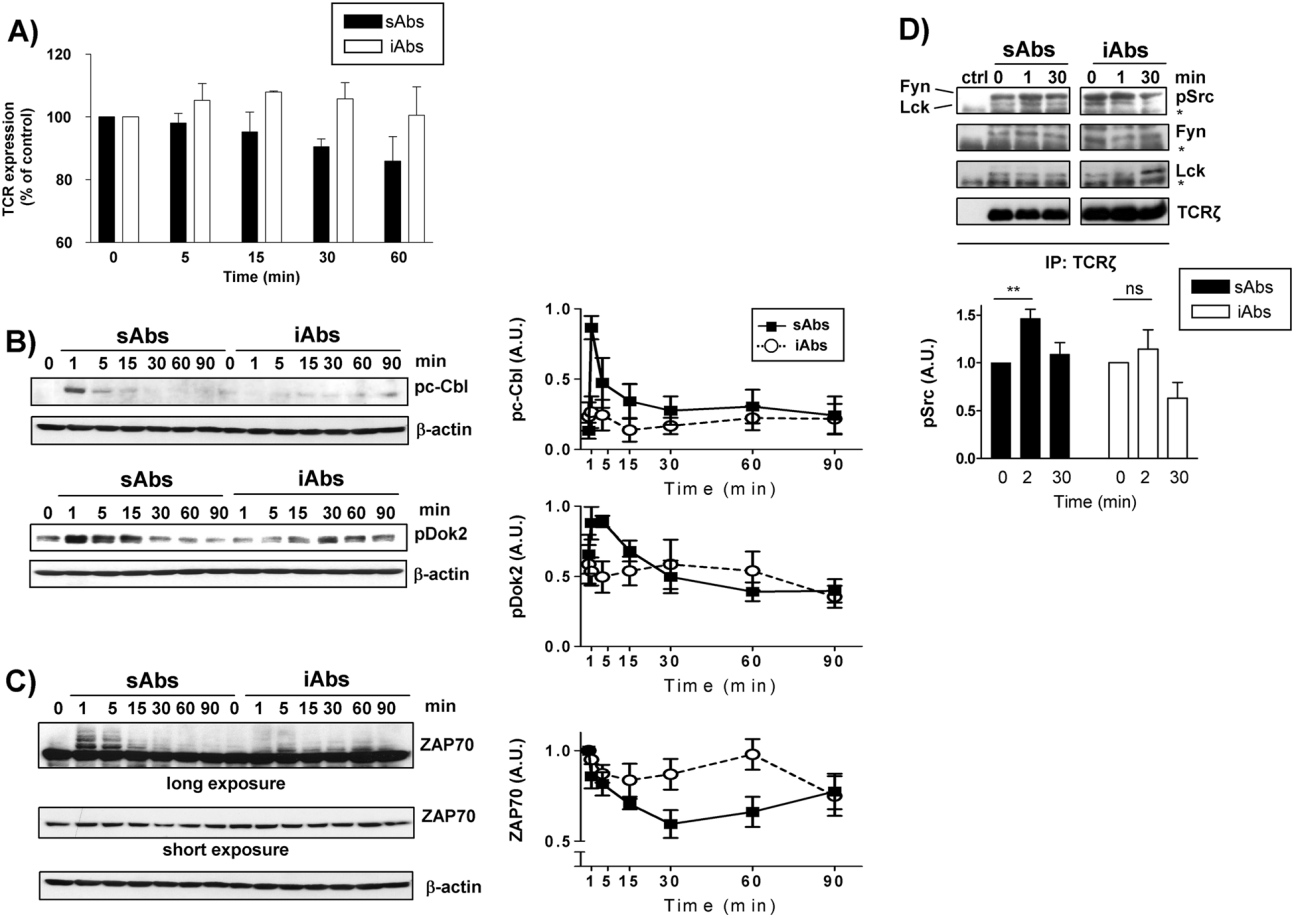
**sAbs, but not iAbs, induce inhibitory feedback loops.** Purified human T cells were treated with either soluble (sAbs) or immobilized (iAbs) CD3xCD28 mAbs for the indicated time points. **A)** Measurement of TCR internalization. The expression of the TCR was assessed by PE-conjugated anti-TCRαβ mAb staining analysis by flow cytometry. Data represent the % of the mean fluorescence intensity (MFI) of the TCR expression relative to time 0 of 4 independent experiments. **B)** The phosphorylation of c-Cbl on Y^731^ and Dok2 on Y^351^ was determined by Western blotting. The phosphorylated c-Cbl and Dok2 bands were quantified using the 1D ImageQuant software and the values were normalized to the corresponding β-actin signal. Data represent the mean of the phosphorylation levels shown as arbitrary units ± SEM of at least 4 independent experiments. **C)** The expression of ZAP70 was determined by Western blotting. Equal loading is shown by reprobing immunoblots with antibodies specific for β-actin. ZAP70 bands were quantified as above. Data represent the mean of the expression levels shown as arbitrary units ± SEM of 6 independent experiments. **D)** Tyrosine phosphorylation of Fyn and Lck in the activation loop was determined by Western blotting using the pSrc (Y^416^) antibody. Bands were quantified using the 1D ImageQuant software and values were normalized to the corresponding total Fyn and Lck signals. Data on the graph represent the mean of the phosphorylation levels shown as arbitrary units ± SEM of 4 independent experiments. Asterisks (*) indicate the Ig heavy chain. Statistical analysis ** P<0.01; ns, not statistically significant.

The data presented above, show that sAbs induced a rapid, but transient TCR-mediated signaling kinetics, which cannot induce productive T-cell response, whereas stimulation with iAbs resulted in a sustained activation of a variety of signaling molecules and led to proliferation. These data indicate that there may be different regulatory mechanisms induced upon sAbs vs. iAbs stimulation. Thus, we next investigated how TCR-mediated signaling is differentially regulated under the two conditions. We hypothesized that a fast internalization of the available TCR molecules upon stimulation with sAbs could provide an explanation for the rapid termination of TCR-mediated signaling. Therefore, we compared the expression levels of the TCR after stimulation with either sAbs or iAbs by flow cytometry. Figure 2A shows that sAbs induce a slow rate of TCR downregulation, which became evident after 30 minutes of stimulation. It is important to note that the majority of the signaling molecules that we have tested reverted to the dephosphorylated/inactive state already 15 minutes after sAbs stimulation (Figure 1). Therefore, termination of TCR-mediated signaling occurs before TCR internalization. On the other hand, the data presented in Figure 2A show that stimulation with iAbs does not reduce, but rather slightly increases TCR levels. This is likely due to the fact that Abs bound to a solid matrix limit TCR internalization, but do not interfere with its transport to the plasma membrane. Moreover, we have previously shown that sustained TCR-mediated signaling and proliferation can occur under conditions of stimulation inducing TCR downregulation [7]. Thus, on the basis of these observations, we exclude that TCR internalization induced by sAbs is the cause of transient signaling.

Having ruled out this possibility, we next focused on the analysis of feedback regulation events, which have been shown to play a crucial role in T-cell activation [11-13]. Proximal negative feedback loops can be activated by the TCR signalosome and can regulate the amplitude, the duration, and the specificity of the signal (reviewed in Acuto et al. [13]). We asked the question of whether the stimulation with sAbs induced the activation of negative regulatory molecules that may terminate signaling, thus resulting in the transient signal observed above. Among the many inhibitory molecules organizing negative regulatory circuits, we decided to focus on c-Cbl, an E3 ubiquitin ligase belonging to the CBL family, and the adaptor protein Dok2, which regulate TCR-mediated signaling through two different mechanisms. Whereas members of the CBL family are involved in the downregulation of signaling molecules via ubiquitination [14], Dok2 and its homolog Dok1 inhibit the activation of signaling pathways by competing for binding to SH2 domains or by recruiting other negative regulators, such as SHIP1 and RasGAP, to the TCR signalosome [15]. The activity of both c-Cbl and Dok2 have been reported to be regulated by tyrosine phosphorylation [15-17] and can be easily monitored by using anti-c-Cbl and anti-Dok2 phosphospecific antibodies, respectively. Figure 2B shows that upon sAbs stimulation, T cells very rapidly and strongly phosphorylated both c-Cbl and Dok2, whereas, treatment of human T cells with iAbs resulted only in a very weak phosphorylation of both molecules.

c-Cbl targets many signaling molecules for degradation, including ZAP70 [18]. Thus, we next tested whether sAbs, in addition to inducing strong c-Cbl phosphorylation, would also induce ZAP70 ubiquitination and degradation. We have previously shown in mouse OT-I T cells that ubiquitination of ZAP70 results in the appearance of ZAP70 bands displaying retarded migration in SDS-PAGE [7]. We checked whether stimulation with soluble CD3xCD28 Abs also resulted in the appearance of ZAP70 bands running at a higher molecular weight in primary human T cells and we found that activation/phosphorylation of c-Cbl upon stimulation with sAbs indeed correlates with retarded ZAP70 migration (Figure 2C). Additionally, the data presented in Figure 2C suggest that stimulation with sAbs also induced ZAP70 degradation. Conversely, stimulation with iAbs did not significantly induce either c-Cbl phosphorylation or retarded migration and degradation of ZAP70 (Figure 2C). Thus, it appears that stimulation with sAbs activates inhibitory feedback loops that may be responsible for terminating TCR-mediated signaling.

In addition to inducing a strong tyrosine phosphorylation of c-Cbl and Dok2, stimulation with sAbs also results in a strong phosphorylation of TCR proximal signaling molecules including TCRζ, ZAP70, and LAT (Figure 1B, 1C). Therefore, we investigated whether sAbs induce a stronger activation of the tyrosine kinases Lck and Fyn compared to iAbs. We immunoprecipitated TCRζ and assessed the level of active Lck and Fyn associated with the TCR. As shown in Figure 2D, sAbs stimulation significantly enhances the level of Lck and Fyn phosphorylated on the activation loop, which is believed to be a sign of an active enzyme. Conversely, this significant increase in Lck and Fyn phosphorylation is not observed upon iAbs stimulation. Hence, the data suggest that, in marked contrast to iAbs, sAbs stimulation enhances Lck and Fyn activation. We postulate that the enhanced activation of Lck and Fyn may result in a stronger tyrosine phosphorylation of downstream molecules (including negative regulators, such as c-Cbl and Dok2), which might imbalance TCR-mediated signaling, thus dampening T-cell activation.

### Sustained activation is regulated by positive feedback loops

**Figure 3:**
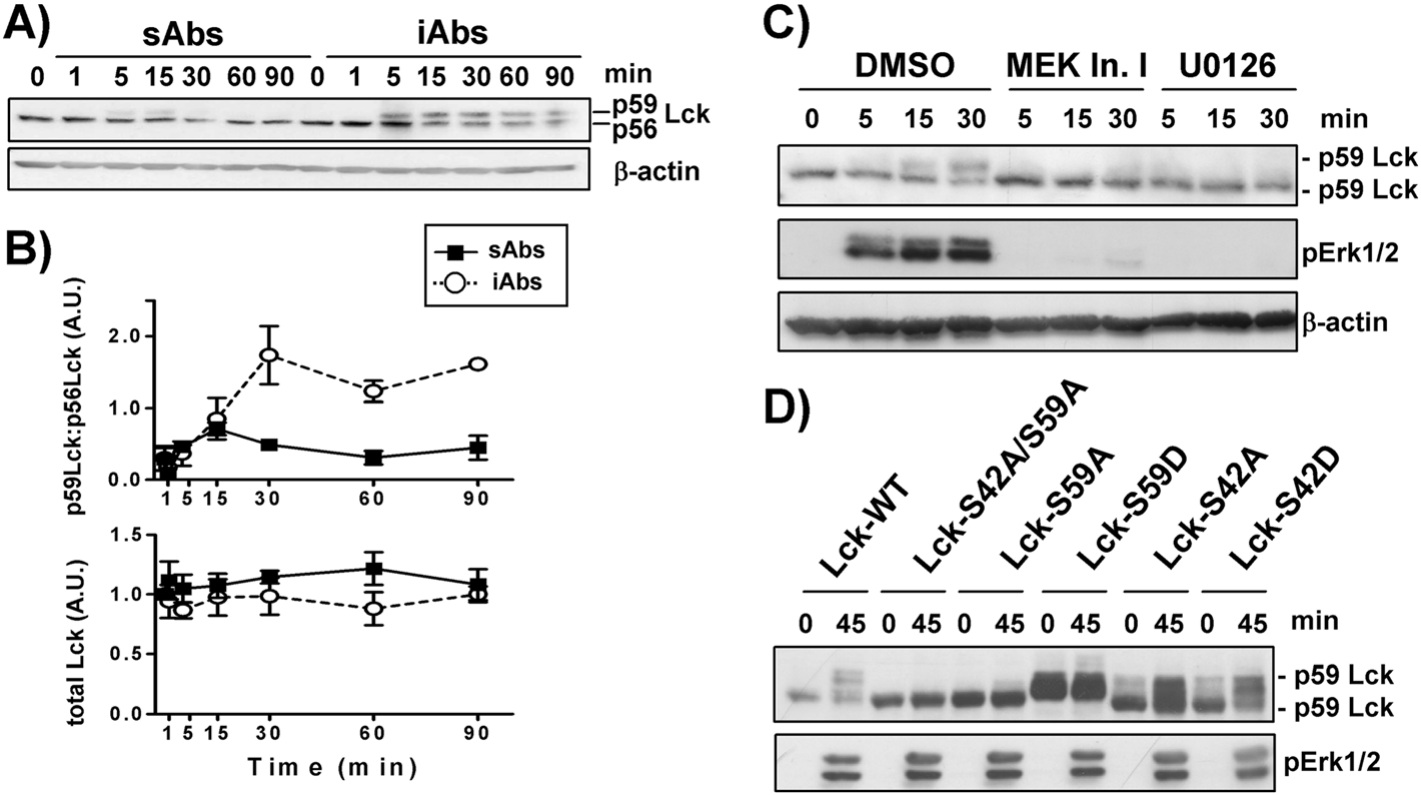
**iAbs induce an Erk-mediated positive feedback loop. A)** Purified human T cells were treated with either soluble (sAbs) or immobilized (iAbs) CD3xCD28 mAbs for the indicated time points. Lck expression was detected in cell lysates by anti-Lck immunoblotting. **B)** Bands corresponding to p56 or p59 Lck were quantified as described in Figure 2. Data represent the ratio of the levels of p56 and p59, and total (p56+p59) Lck shown as arbitrary units ± SEM of 5 independent experiments. **C)** Purified human T cells were treated with immobilized (iAbs) CD3xCD28 mAbs for the indicated time periods in the presence or absence of MEK Inhibitor I or U0126. Samples were analyzed by Western blotting using the Abs indicated. One representative immunoblot of 4 independent experiments is shown. **D)** J.CaM1.6 cells were transfected with various constructs carrying different mutations (S42A, S42D, S59A, S59D, S42A/S59A). After transfection, cells were either left unstimulated or stimulated with iAbs for 45 min. Samples were analyzed by Western blotting using the indicated Abs. One representative experiment of five independent experiments is shown.

We next investigated whether positive feedback loops may be triggered by iAbs, thus leading to sustained activation of TCR-mediated signaling. In particular, we explored the regulatory circuit involving Lck phosphorylation by activated Erk [11]. This model is based on observations showing that the Erk-mediated phosphorylation of Lck on serine 59 alters Lck mobility and the ability of the SH2 domain of Lck to bind phosphotyrosines [19-21]. Stefanova et al. further demonstrated that Erk-mediated phosphorylation of Lck prevents SHP-1 binding, thus interfering with SHP-1-mediated Lck inactivation [11]. According to this model, active Erk would feedback to Lck to sustain signaling. To assess whether stimulation with iAbs triggers this Erk-mediated positive feedback loop, T cells were stimulated with iAbs and sAbs and the phosphorylation of Lck on S^59^ was detected by the appearance of a new Lck band running at 59 kDa by Western blot [11]. As shown in Figure 3A and B, stimulation of T cells with iAbs clearly resulted in the formation of p59 Lck (up to 50% of total Lck), whereas this shift in the molecular weight of Lck was barely detectable upon sAbs treatment.

To demonstrate that the appearance of p59 Lck indeed depends on Erk-mediated phosphorylation, T cells were stimulated with iAbs for 30 min in the presence or absence of U0126 or MEK Inhibitor I, inhibitors of the Erk activator MEK. This treatment has previously been shown to abolish the conversion of Lck to the p59 form [11]. In agreement with these observations, we also found that treatment of iAbs-stimulated T cells with U0126 or MEK Inhibitor I completely abolished both Erk activation and the shift of Lck to the p59 form (Figure 3C). We next tested whether the molecular shift of Lck upon iAbs stimulation is indeed induced by phosphorylation of S^59^. To assess this issue, we took advantage of Lck constructs carrying S to D and S to A mutations at this position, which mimic constitutive phosphorylation or prevent phosphorylation, respectively. We used the following mutants S59D, S59A, S42D, S42A, and S42A/S59A, which were expressed in the Lck-deficient Jurkat T-cell line J.CaM1.6. As shown in Figure 3D, mutations of S^42^ do not affect the mobility shift of Lck either in unstimulated or iAbs stimulated cells. Conversely, the S59D mutation results in a constitutive shift to p59 Lck, thus indicating that phosphorylation on this site plays a major role in the regulation of Lck mobility. Accordingly, the S59A substitution, which results in a non-phosphorylatable mutant, prevents the generation of the 59 kDa form of Lck upon iAbs stimulation (Figure 3D). In summary, these data demonstrate that Erk-mediated phosphorylation of Lck at S^59^ results in its retarded mobility on SDS-PAGE.

**Figure 4:**
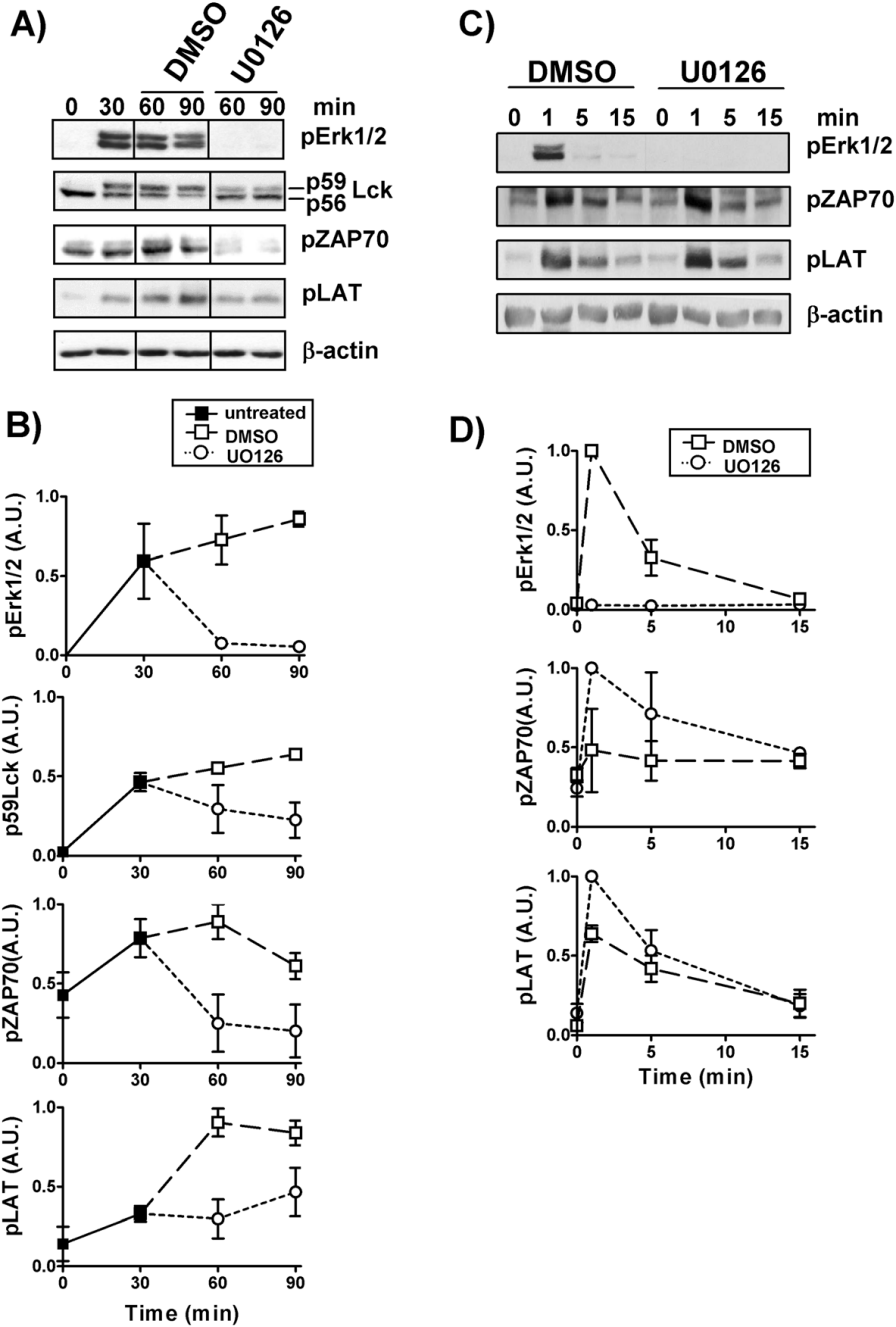
**An Erk-Lck feedback loop regulates TCR-mediated signaling. A)** Purified human T cells were treated with iAbs alone for 30 min and then either DMSO or the MEK inhibitor U0126 was added and incubated for an additional 30 to 60 min. Samples were analyzed by Western blotting using the indicated Abs. **B)** Bands in A) were quantified and the values were normalized as described. Graphs show the mean of the phosphorylation levels of Erk1/2, ZAP70, and LAT or the level of p59 Lck as arbitrary units ± SEM of 4 independent experiments. **C)** Purified human T cells were preincubated either in the presence of DMSO or the MEK inhibitor U0126 and subsequently stimulated with sAbs for the indicated time points. Samples were analyzed by Western blotting using the indicated Abs. **D)** Bands in **C)** were quantified as described above and the data from at least two independent experiments are shown.

To check whether the inhibition of Erk-mediated Lck phosphorylation also resulted in a reduction of its activity, we investigated phosphorylation levels of downstream signaling molecules that are substrates of Lck, such as the tyrosine kinase ZAP70 and the adaptor protein LAT whose phosphorylation depends on ZAP70. T cells were stimulated for 30 min with iAbs. Subsequently, Erk activity was blocked by the addition of the MEK inhibitor U0126. The data presented in (Figure 4A, B) show that the phosphorylation of both ZAP70 and LAT is reduced upon MEK inhibition, thus indicating that Erk-mediated Lck phosphorylation may enhance its response. Conversely, treatment of sAbs-stimulated T cells with the MEK inhibitor reduced Erk phosphorylation, as expected, but not ZAP70 or LAT phosphorylation (Figure 4C, D).

Collectively, these data suggest that stimulation with iAbs activates an Erk-mediated positive feedback loop which is required for proper T-cell response and proliferation. Importantly, the regulatory circuit induced by iAbs seems to mimic a previously described mechanism that is induced in T cells upon physiological stimulation [11].

### Enhancement of Src kinases phosphorylation converts sustained into transient signal

**Figure 5:**
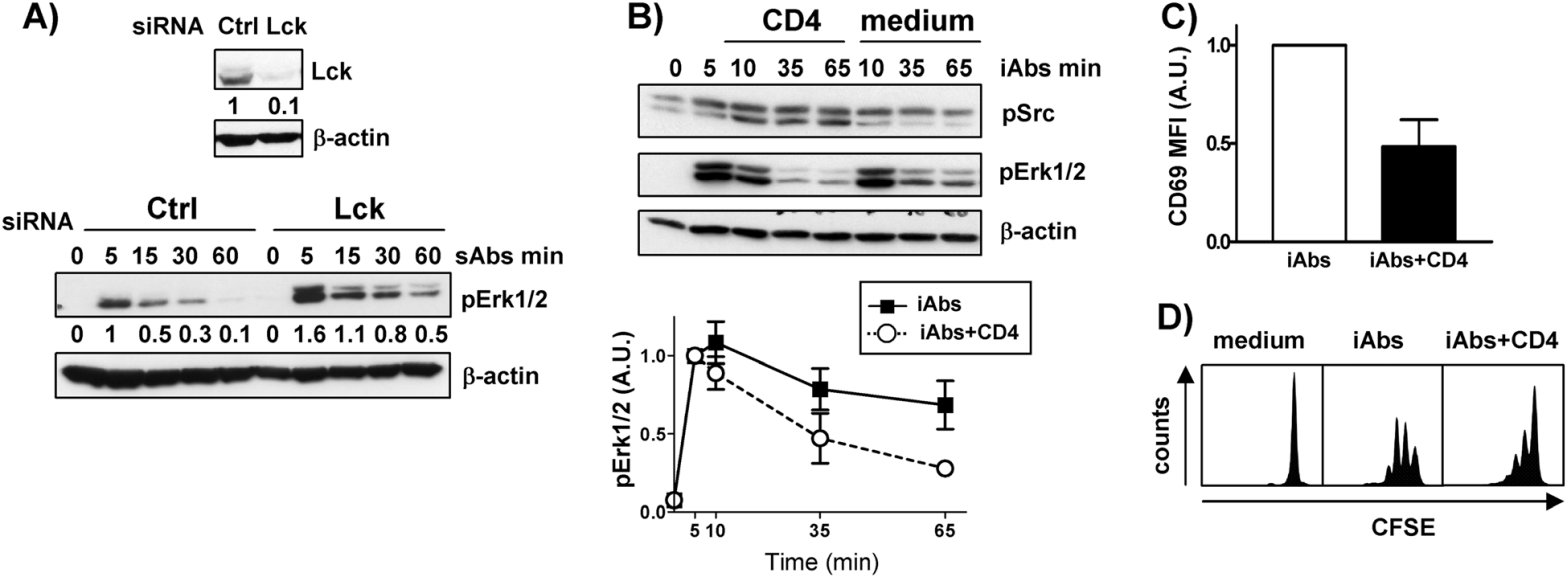
**Lck phosphorylation correlates with decreased T-cell activation. A)** Jurkat T cells were transfected with Lck siRNA duplex or siRNA control (Ctrl) and cultured for 48 h. Subsequently, cells were stimulated with soluble CD3 mAbs (clone OKT3) for the indicated times. Cell lysates were analyzed by immunoblotting using the indicated Abs. Immunoblot verifying Lck downregulation is shown. One representative experiment of three independent experiments is shown. **B)**-**D)** Purified human CD4^+^ T cells were treated with immobilized (iAbs) CD3xCD28 mAbs in the presence or absence of cross-linked CD4 mAb as indicated. **B)** The phosphorylation levels of Erk1/2 and Src kinases were determined by Western blotting. The phospho-specific bands were quantified using the 1D ImageQuant software and the values were normalized to the corresponding β-actin signal. Data on the graph represent the mean of the phosphorylation levels shown as arbitrary units ± SEM of 3 independent experiments.** C) **24h after stimulation, the activation of CD4^+^ T cells was analyzed by staining with CD69 and flow cytometry. Data on the graph represent the mean of the expression levels shown as arbitrary units ± SEM of 3 independent experiments. **D)** CD4^+^ T cells were labeled with CFSE and stimulated as indicated. Proliferation was assed after 72h by analyzing CFSE content on a LSRFortessa. One representative experiment of 3 independent experiments is shown.

The data presented above suggest that sAbs and iAbs induce qualitatively different signals and feedback regulation which are translated into distinct cellular responses. How the cell senses the quality of the signal is not yet fully understood. Our data suggest that sAbs induce stronger Src kinases activation and a stronger tyrosine phosphorylation pattern compared to iAbs stimulation (Figure 1). These observations may suggest that Src kinases are involved in deciphering the nature of the signal. To test the contribution of Lck, the major Src kinase in T cells, in the regulation of signaling dynamics, we suppressed its expression by RNAi in Jurkat T cells and evaluated the effects on Erk activation. Figure 5A shows that cells expressing low amount of Lck displayed prolonged Erk1/2 activation. These observations are in line with previous studies showing that knockdown of Lck in Jurkat and primary human T cells prolonged Erk phosphorylation and transcriptional activation [22,23].

We next decided to investigate whether strong phosphorylation of Lck and Fyn may convert a sustained into a transient signal. To this aim, CD4^+^ primary human T cells were stimulated with iAbs for a short time period and subsequently CD4 was cross-linked using soluble anti-CD4 mAbs. It is known that CD4 crosslinking results in *trans*-phosphorylation of Lck, thus strongly enhancing its activity. As presented in Figure 5B, CD4 crosslinking indeed resulted in a strong induction of Lck phosphorylation measured using an anti-pY^416^Src antibody. Most importantly, enhanced Lck phosphorylation paralleled with a significant reduction in Erk phosphorylation (Figure 5B). Accordingly, we found that also CD69 expression and proliferation were strongly reduced upon CD4 crosslinking (Figure 5C, D). These data suggest that strong Src-family kinase activity may result in the activation of inhibitory signals suppressing T-cell activation.

**Figure 6:**
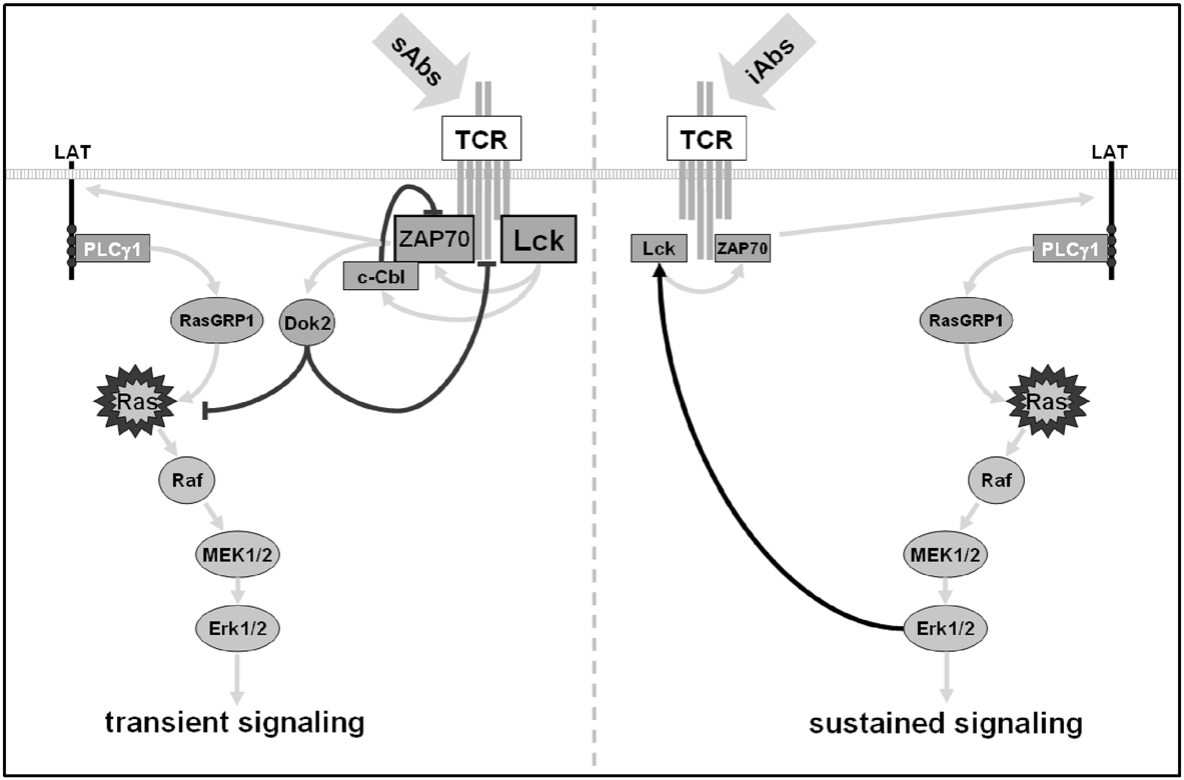
**Feedback regulation of TCR-mediated signaling.** sAbs stimulation triggers strong phosphorylation of Src kinases, such as Lck, and leads to strong activation of downstream signaling pathways. In addition to the activation of positive regulators, sAbs also induce inhibitory molecules (c-Cbl, Dok2), which might imbalance TCR-mediated signaling, thus rapidly terminating T-cell activation (left side). On the other hand, iAbs stimulation results in the activation of an Erk-Lck positive feedback loop, which is required to sustain signaling (right side).

In summary, we have shown that stimulation with iAbs induces different feedback regulation than sAbs treatment (Figure 6). sAbs lead to strong and rapid activation of Src kinases and subsequently to the phosphorylation of inhibitory molecules (e.g. c-Cbl, Dok2), which terminate signaling. On the other hand, iAbs induce only slight increase in kinase activity and an Erk-Lck positive feedback loop, which may be required to prevent rapid Lck dephosphorylation by SHP-1 or other phosphatases, and therefore lead to sustained activation.

## Discussion

Signaling through a variety of plasma membrane-associated receptors leads to cell decision processes such as cell proliferation, differentiation, survival, and motility. Considerable evidence suggests that the magnitude and the duration of a signal determine the functional outcome. As little is known on the mechanisms regulating signaling kinetics correlating with cellular responses in T cells, we have analyzed TCR-mediated signaling under conditions leading to either T-cell unresponsiveness or to proliferation. We employed sAbs and iAbs stimulation which induce qualitatively different signals and T-cell responses [8]. We found striking differences in TCR signaling kinetics and feedback regulation. Indeed, under proliferation-inducing conditions, TCR-mediated signaling is prolonged by a positive feedback loop involving Erk and Lck. Conversely, stimulation with sAbs strongly activates inhibitory molecules that likely terminate signaling. These observations are in agreement with the model proposed by Acuto et al., that the signal amplitude and kinetics in double positive thymocytes depend on the type of the applied stimulus [13]. Here, we show that a similar principle may apply also to mature T cells.

An important question that needs further investigations is how signals with a common origin at the TCR are split to activate different effector molecules. During thymocyte development, it has been proposed that a molecule or complex functioning as “signal splitter” senses the intensity of signals emanating from the TCR and discriminates between negatively and positively selecting ligands [24]. However, such a molecule has not yet been identified in immature T cells. We propose that Lck may function as “signal splitter” in mature T cells directing signals emanating from the TCR toward unresponsiveness if the signal is at high intensity (i.e. in case of stimulation with sAbs, which induce strong Src kinase activation). This safety mechanism could be set in motion in case of an inappropriate stimulation that could lead to T-cell hyperactivation and the development of autoimmunity. The idea that the molecular switch is located at the apical part of the cascade could represent an advantage of the system. In fact, the termination of the signal at a membrane proximal level will require only the activation of a limited number of downstream inhibitory pathways to efficiently stop activation. In case of an appropriate stimulus, such as iAbs, Lck activity is not substantially increased over the basal level. As proposed by Nika et al., the pool of constitutively active Lck is sufficient to initiate the signaling cascade [25]. This weaker signal will in turn activate positive feedback loops which enhance the strength and prolong the activation of more distal signaling cascades, thus culminating in proliferation.

How Lck senses the characteristic of the stimulus triggering the TCR, which will in turn result in the generation of the appropriate cellular program, is not yet known. However, when we compared sAbs vs. iAbs, we found that Lck undergoes different phosphorylation events. Whereas sAbs enhance phosphorylation of Lck at Y^394^, which is believed to enhance its kinase activity, iAbs induce phosphorylation of Lck at S^59^. We propose that phosphorylation at Y^394^ induced by sAbs results in a hyper-phosphorylation of downstream signaling molecules that disturbs the equilibrium between positive and negative regulators of TCR-mediated signaling, favoring inhibitory signals (like c-Cbl) that shutdown T-cell activation. This hypothesis is also supported by our observations and previously published data showing that suppression of Lck expression by RNAi strongly impaired the activation of the inhibitory molecules SHP-1 and c-Cbl and also prolonged downstream signaling (i.e. pErk, NFAT/AP-1) induced by soluble CD3 stimulation [22,23]. Thus, strong Lck activation may have inhibitory effects on T-cell activation. On the other hand, phosphorylation on S^59^ may be required to prevent rapid deactivation by SHP-1. Moreover, if Lck becomes strongly active, this would in turn shutdown signaling as in the case of sAbs stimulation. Interesting in this regard, we found that iAbs stimulation not only enhances phosphorylation on S^59^ but concomitantly reduces phosphorylation of Y^394^ at later time points after activation (Figure 2D and our unpublished results).

We also found that crosslinking of CD4 in cells undergoing activation dampen T-cell responses. We propose that a strong activation of Src kinases induced upon CD4 crosslinking may triggers inhibitory feedback loops in a similar manner to sAbs. Interestingly, when anti-CD4 is immobilized together with anti-CD3 on microbeads, T-cell activation is enhanced compared to stimulation with anti-CD3 alone. Under this condition, an enhanced Src kinase phosphorylation was not observed [8]. Previous observations had shown that crosslinking of CD4 before stimulation also impaired T-cell activation [26]. This mechanism has been implicated in T-cell depletion occurring during HIV infection [27]. Our data suggest that crosslinking of CD4 by gp120 and anti-gp120 antibodies may shutdown T-cell activation also during immune responses in HIV-infected patients, thus contributing to immunodeficiency.

In addition to Lck, we have found major differences in the regulation of Erk activation between sAbs and iAbs. It has been previously shown that Erk activity in cytotoxic mouse T lymphocytes after stimulation with immobilized antibodies depends on nPKCs, whereas, sAbs stimulation activates Erk also via cPKCs [28]. Thus, TCR-mediated Erk activation under condition of stimulation correlating with proliferation appears to be not only quantitatively, but also qualitatively different from that induced by sAbs.

## Conclusions

In summary, we show that TCR-mediated signaling kinetics and feedback regulation under proliferation-inducing conditions (iAbs) are markedly different from those leading to unresponsiveness (sAbs) and we provide some potential mechanistic insights that may explain this differential behavior. We hope that the comparative analyses presented here will inspire further studies aimed at dissecting the spatio-temporal regulation of T-cell activation.

## Methods

### Human Ethics

Approval for these studies involving the analysis of TCR-mediated signaling in human T cells was obtained from the Ethics Committee of the Medical Faculty at the Otto-von-Guericke University, Magdeburg, Germany with the permission number [107/09]. Informed consent was obtained in writing in accordance with the Declaration of Helsinki.

### Cell purification

Peripheral blood mononuclear cells were isolated by Ficoll gradient (Biochrom) centrifugation of heparinized blood collected from healthy volunteers. Total population of human T cells or CD4^+^ subpopulation were further purified by non-T cell depletion using T cell isolation kits (Miltenyi Biotec). The purity of T cells, determined by flow cytometry, was usually more than 96%.

### T-cell stimulation

After isolation, T cells were cultured overnight in RPMI 1640 medium containing 10% FCS (PAN Biotech) and 2 μg/ml Ciprobay (Bayer Schering Pharma). Successively, T cells were stimulated with either soluble or immobilized mAbs as follows. For soluble Ab stimulation, 2x10^6^ cells were loaded with 10 μg/ml biotinylated anti-human CD3 (clone UCHT1, eBioscience) in combination with 10 μg/ml biotinylated anti-human CD28 (clone CD28.2, eBioscience) mAbs in 100 μl RPMI 1640 for 15 min on ice. After washing, receptors were cross-linked by adding 25 μg/ml NeutrAvidin™ (Pierce). For microbead stimulation, SuperAvidin™-coated polystyrene microspheres (Ø~10 μm, Bangs Laboratories) were coated with biotinylated CD3 in combination with CD28 mAbs (10 μg/ml each) for 30 min at 37°C in PBS. Antibody-coated microbeads were washed twice with PBS, resuspended in RPMI 1640 and incubated with T cells in a 1:1 ratio. For stimulation of pre-activated cells 10 μg/ml of purified IgM anti-human CD4 (clone MEM16, kindly provided by V. Horejsi, Academy of Sciences of the Czech Republic, Czech Republic) was used. For Jurkat T cell stimulation soluble CD3 mAbs (clone OKT3) was used.

Stimulations in the presence of either the MEK inhibitor I, U0126 (Cell Signaling Technology) or DMSO (Sigma-Aldrich) were performed by pre-incubating T-cells for 30 min with 10 μM of the compounds before stimulation with mAbs. For indicated microbead stimulation, 10 μM of either UO126 or DMSO were added 30 min after stimulation.

### Cell transfections

The Jurkat T-cell line and Lck-deficient variant of the Jurkats (J.CaM1.6) were maintained in RPMI 1640 medium supplemented with 10% FCS (PAN Biotech) and antibiotics at 37°C and 5% CO_2_. For cell transfection, we used pBos expression plasmid encoding various Lck constructs (S42A, S42D, S59A, S59D, S42A/S59A). For RNAi experiments siRNA Lck duplex containing 21 nucleodites was purchased from Life Technologies. The sequences were as follows, sense: 5’–UAACCAGGUUGUCUUGCAGUG–3 antisense: 5’–CUGCAAGACAACCUGGUUAUC–3’. As a negative control we used a Renilla Luciferase siRNA duplex 5´–CCAAGUAAUGUAGGAUCAATT–3’. To achieve efficient transfection, Jurkat T cells were electroporated using the Gene Pulser II (Bio-Rad) as previously described [29]. 48 h after electroporation cells were collected, stimulated with iAbs or sAbs as indicated, and processed for Western blotting.

### Immunoprecipitation

Primary human T cells (3×10^7^) were either left untreated or stimulated with sAbs or iAbs for the indicated periods of time. Cells were lysed in 1% Brj58 or 1% lauryl maltoside (N-dodecyl β-maltoside), 1% IGEPAL CA-630, 1 mM Na_3_VO_4_, 1 mM PMSF, 10 mM NaF, 10 mM EDTA, 50 mM Tris pH 7.5, and 150 mM NaCl, and cleared by centrifugation. TCRζ chains were immunoprecipitated with agarose-conjugated CD3ζ (Santa Cruz Biotechnology) antibody followed by recombinant protein A-agarose beads (Santa Cruz Biotechnology) at 4°C overnight. After washing, TCRζ immunoprecipitates were resolved by SDS-PAGE, transferred to a nitrocellulose membrane (Amersham), and analyzed by immunoblotting with the indicated antibodies.

### Western blotting

T cells were lysed in buffer containing 1% lauryl maltoside (N-dodecyl β-maltoside), 1% IGEPAL CA-630, 1 mM Na_3_VO_4_, 1 mM PMSF, 10 mM NaF, 10 mM EDTA, 50 mM Tris pH 7.5, and 150 mM NaCl. Post-nuclear lysates were separated by SDS-PAGE and transferred onto nitrocellulose membranes (Amersham). Membranes were probed with the indicated primary antibodies and the appropriate HRP-conjugated secondary antibodies (Dianova) and developed using the ECL detection system (Amersham). The following antibodies were used for Western blotting in this study: anti-phospho(p)-T^202^/Y^204^ Erk1/2, anti-pY^319^ZAP70, anti-pY^171^LAT, anti-pY^783^PLCγ1, anti-pS^338^-c-Raf, anti-pS^217/221^MEK1/2, anti-pS^380^p90RSK, anti-pY^731^-c-Cbl, anti-pY^351^-p56Dok2, anti-pY^416^Src (all from Cell Signaling Technology), anti-Lck (from BD Transduction laboratories), anti-Lck (from Epitomics), anti-Fyn (Fyn01, kindly provided by Vaclav Horejsi), anti-ZAP70, anti-CD3ζ (Santa Cruz Biotechnology), anti-pTyr (clone 4G10)-HRP conjugate (Millipore), and anti-β-actin (clone AC-15) (Sigma-Aldrich). For quantifications of the Western blots, the intensity of the detected bands was acquired using the Epson Perfection V700 Photo Scanner and analysis was performed using 1D ImageQuant software (Kodak). Unless indicated otherwise, β-actin was used as a loading control (typical loading error in the experiment: ± 13%).

### *In vitro* assays

Proliferation experiments were carried out in 96-well plates (Costar). Purified human T cells or CD4+ subpopulation were labeled with 2.5 μM CFSE (Molecular Probes) for 10 min at 37°C. After washing, 2×10^5^ cells were seeded in a total volume of 200 μl to each well and cultured in RPMI (supplemented with 10% FCS and antibiotics). T cells were either left unstimulated or stimulated with soluble or immobilized CD3×CD28 mAbs in the presence or absence of soluble CD4 as indicated. T cells were cultured for 72 h at 37°C, 5% CO_2_. Proliferation was assessed by CFSE dilution using a BD LSRFortessa, FACSDiva Software 6.1.3 (BD Biosciences), and FlowJo 7.6.5 (Tree Star, Inc.).

To determine the efficiency of T-cell activation, T cells were stimulated as described above. After 24h, T cells were stained with PE-labeled mAbs against CD69 (BD Biosciences) and analyzed by flow cytometry.

### TCR internalization

To determine TCR internalization, 1×10^6^ cells were stimulated with sAbs or iAbs as mentioned above at 37°C for 0–60 min. Cells were stained with PE-conjugated TCRαβ mAb (BD Biosciences) for 15 min at 4°C and analyzed by flow cytometry.

## Abbreviations

TCR: T cell receptor; sAbs: Antibodies cross-linked in solution; iAbs: Immobilized on microbeads.

## Competing interests

The authors declare that they have no competing financial and non-financial interests.

## Authors’ contributions

MP carried out the biochemical studies, performed the proliferation assays and the statistical analysis; BA participated in the design of the study and carried out the biochemical studies; AVR and BSK carried out the biochemical studies; VW participated in the analysis of Lck mutants; JAL and BS participated in the design of the study and helped to draft the manuscript; LS performed the statistical analysis, conceived and coordinated the study, and drafted the manuscript. All authors read and approved the final manuscript.

## Authors’ information

B.A. and M.P. contributed equally to this work. BA present address, Diabetes Centre, Department of Medicine Innenstadt, Ludwig-Maximilian University, 80336 Munich, Germany. BSK present address, Vectorology and Experimental Gene Therapy, University of Rostock, 18057 Rostock, Germany, VW present address, MERCK, Munich, Germany. JAL present address, Department of Nephrology, Hypertension, Diabetes, and Endocrinology Otto-von-Guericke University, 39120 Magdeburg Germany, JAL and BS are members of SYBILLA [EU7FP] and the Magdeburg Center for System Biology (MaCS).
